# Early-Onset Multiple Sclerosis With Frequent Relapses: A Challenging Diagnosis With a Less Favorable Prognosis

**DOI:** 10.7759/cureus.13963

**Published:** 2021-03-18

**Authors:** Vijayakumary Thadchanamoorthy, Kavinda Dayasiri

**Affiliations:** 1 Clinical Sciences Department, Faculty of Health Care Sciences, Eastern University, Batticaloa, LKA; 2 Paediatrics, Base Hospital Mahaoya, Mahaoya, LKA

**Keywords:** multiple sclerosis, hemiparesis, methyl prednisolone

## Abstract

Pediatric multiple sclerosis (MS) is a rare demyelinating disease of the brain, spinal cord, and optic nerve caused by immune modulators mediating against the neuronal axons of the central nervous system. MS is usually characterized by a series of neurological events, without any features of encephalopathy, separated in time and space. The complications arise from the permanent degeneration of the nerves. This condition can be diagnosed based on International Pediatric Multiple Sclerosis Study Group diagnostic criteria, and there is no definitive treatment for MS. We report the case of a male child who was diagnosed with MS at the age of six years when he presented with right hemiparesis and visual impairment. Subsequently, he had multiple relapses with varied neurological presentations, and each relapse was treated with methylprednisolone.

## Introduction

Multiple sclerosis (MS) is a chronic demyelinating condition of the central nervous system characterized by a relapsing-remitting course of neurological events separated in time and space [1,2]. MS can present as hemiparesis or paraparesis [3], optic neuritis [4], brain stem dysfunction [3], sensory loss, and ataxia; it can also manifest with bowel and bladder symptoms [5]. The immune system dysregulation involving T and B lymphocytes triggers inflammation, axonal demyelination, axonal loss, and regeneration within both white and gray matter, which causes the relapsing-remitting pattern of the disease [6].

MS is rare in Asian countries, with an incidence rate of 0.8-2/100,000 people [7]. In most cases, patients will not develop symptoms until they reach young adulthood. MS is more commonly seen in females than males, with a female-to-male ratio of 2:1 [8]. MS is very rarely reported in early childhood [9]. In this report, we present the case of a six-year-old South Asian boy who experienced multiple relapses of MS with varied neurological presentations.

## Case presentation

**Figure 1:**
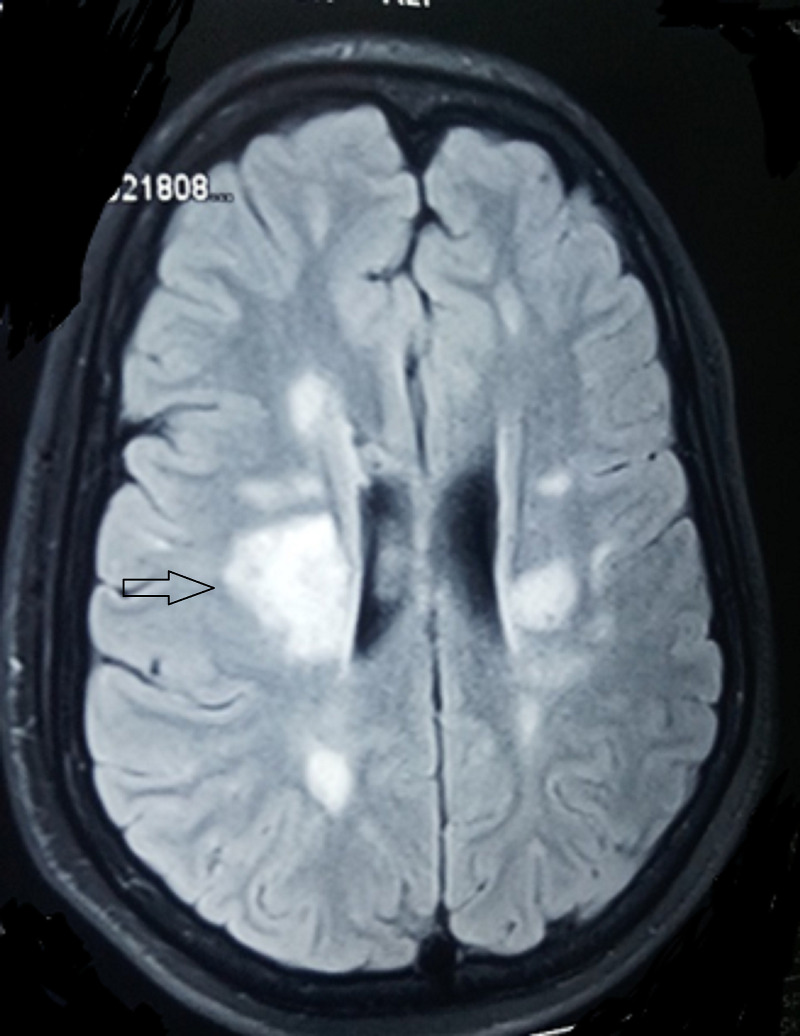
Hyperintense white matter lesions (arrow) on T2-weighted MRI brain supportive of multiple sclerosis diagnosis MRI: magnetic resonance imaging

**Figure 2:**
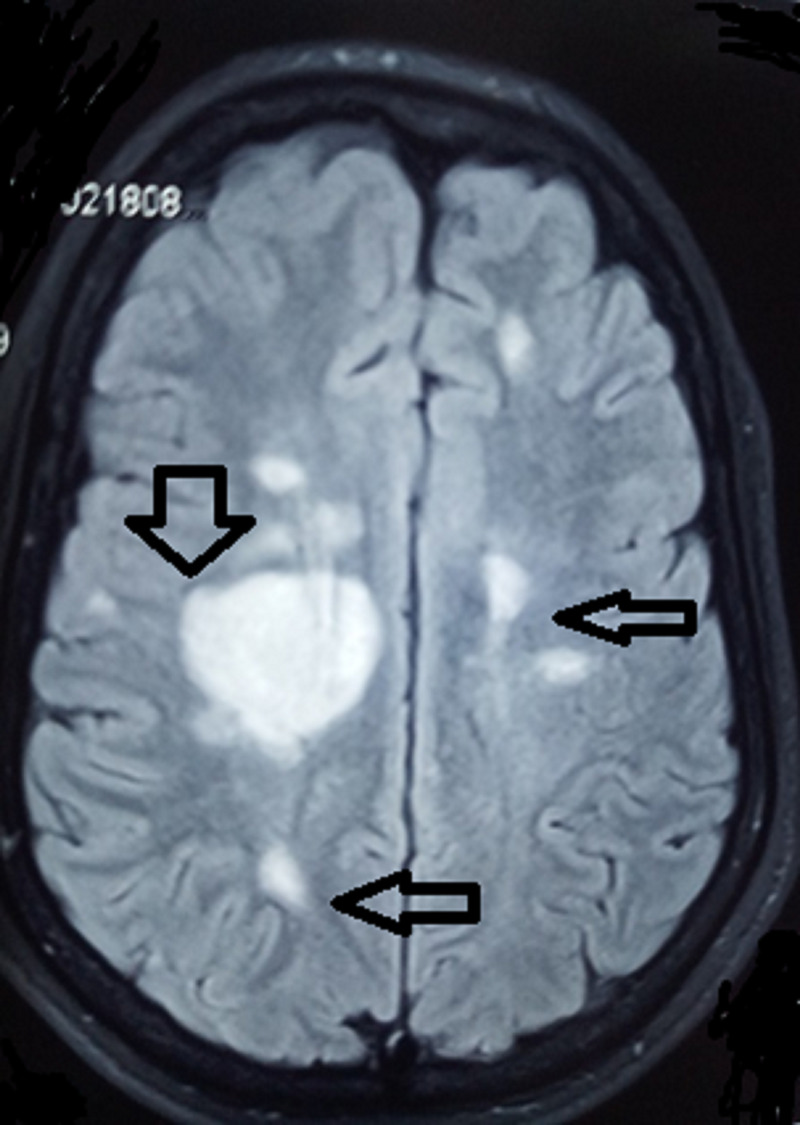
Multiple hyperintense lesions (arrows) on T2-weighted MRI brain supportive of multiple sclerosis diagnosis MRI: magnetic resonance imaging

A six-year-old boy presented to the pediatric ward with left-sided hemiparesis and facial nerve weakness of one month's duration. It was examined with blood and biochemical investigations including MRI brain with contrast. The MRI showed multiple well-defined focal lesions bilaterally in the parietal region, predominantly on the right side. Other investigations were normal. The findings of MRI brain and spinal cord were suggestive of MS and the child was treated with methylprednisolone followed by oral prednisolone. Subsequently, the patient experienced numerous relapses with multiple episodes of limb weakness and facial nerve weakness. MRI brain and spinal cord was repeated during each episode and showed multiple new lesions, which had not been found in previous studies. The pattern of clinical and neuroradiological features was compatible with MS (as per the International Pediatric Multiple Sclerosis Study Group criteria for pediatric multiple sclerosis and immune-mediated central nervous system demyelinating disorders). During relapses, the child was treated with methylprednisolone. Figures 1, 2 show multiple hyperintense white matter lesions on T2-weighted MRI, which were supportive of a diagnosis of MS.

Subsequently, the patient was treated once with intravenous interferon due to frequent relapses and increased severity. However, owing to the side effects of the drug (fever with chills and headache), it was discontinued. During the latest episode at 13 years of age, he was admitted following an episode of right-sided hemiparesis, visual impairment, difficulty in walking and swallowing for three weeks, and fever for one day. It was managed as a relapse of MS and bacterial infection as the child had a high fever with high inflammatory markers. He was treated with intravenous methylprednisolone and intravenous antibiotics. Following the commencement of treatment, he improved gradually. He was discharged with oral prednisolone, and his physiotherapy and occupational therapy were continued. The patient is currently being followed up at the multidisciplinary clinic.

## Discussion

MS is a rare disease in the pediatric population with only 1.7-5.6% of patients showing symptoms before the age of 18 years [10]. Its prevalence varies from very low in the peri-equatorial region to high in temperate regions, and this pattern raises a concern related to limited sunlight exposure and vitamin D insufficiency as potential risk factors for MS [11].

MS is a chronic inflammatory demyelinating condition of the central nervous system, which is characterized by myelin loss, axonal degeneration, and often progressive neurological dysfunction. The irregularity of the immune response in the central nervous system could occur both as a primary component of MS autoimmunity and as a response to tissue injury (6).

The initial occurrence of central nervous system inflammatory demyelination may present as an isolated monophasic illness or may represent the first attack of MS. Many studies have described the clinical and radiological risk factors for MS development after the first attack of demyelinating disease [12,13]. The presence of oligoclonal bands in cerebrospinal fluid (CSF), past Epstein Barr viral infection, periventricular lesions, hypointense lesions on T1, and lesions of the corpus callosum have been reported to be the best predictors of the clinical evolution of the condition [13]. However, no single clinical feature or radiological risk factor distinguishes acute demyelinating encephalomyelitis (ADEM) from MS. The relapsing and remitting course becomes secondarily progressive later in adulthood. Long-term follow-up often reveals the progressive nature of the disease.

When a child presents with hemiparesis or paraparesis, visual disturbance, and ataxia, it is challenging to differentiate MS from ADEM and neuromyelitis optica [14]. But during an acute presentation, in the absence of clear evidence of an infectious cause, the MRI findings should define the distribution of the demyelinating inflammatory process in the central nervous system. Changes in the MRIs are supportive but not diagnostic and often reveal multifocal, diffuse, and hyperintense T2 white matter lesions [15]. The gray matter is frequently involved and MRI of the spinal cord may also show intramedullary lesions. A diagnosis of MS should be considered in cases with recurrent demyelination. Callen et al. have described the radiological criteria [16] that can be used to distinguish MS from ADEM, and they include any two of the three following features: (1) absence of a diffuse bilateral lesion pattern, (2) presence of black holes, and (3) presence of two or more periventricular lesions.

There may be non-remitting neurological lesions affecting vision, sensory and motor functions, and bowel and bladder control in MS. Children with chronic MS will also develop cognitive impairment, which could affect their academic progress. Our patient is an average student in terms of scholastic performance at school and until now, there have been no signs of cognitive and intellectual impairment in him.

The relapses are treated with methylprednisolone 20-30 mg/kg/day for three to five days with or without oral prednisolone taper-off. Intravenous immunoglobulin might be an alternative treatment option for those who have contraindications for corticosteroids or in whom corticosteroids are less effective [17]. In some children with severe symptoms who do not respond to the first course of high-dose corticosteroids, a second pulse may be useful.

Despite undergoing treatment for MS, pediatric patients can acquire an irreversible disability at a younger age compared to adults who take a longer time to develop the same (20-30 years) [18]. A less favorable prognosis with neurodisability is expected in children with short intervals between attacks [19] and frequent relapses in the first year of the onset of the disease.

## Conclusions

We presented the case of a male child with early-onset MS. The prognosis is likely not favorable for this patient due to the early onset of the disease, short interval between attacks, and frequent relapses during the first year of the disease. However, the early diagnosis of MS in this patient enabled us to initiate prompt and appropriate treatment, thereby delaying long-term permanent physical disability and intellectual impairment.
